# Understanding mental health promotion in organized leisure communities for young people: a realist review

**DOI:** 10.3389/fpubh.2024.1336736

**Published:** 2024-04-17

**Authors:** Amalie Oxholm Kusier, Thilde Risager Ubbesen, Anna Paldam Folker

**Affiliations:** The Research Department for Health and Social Context, National Institute of Public Health, University of Southern Denmark, Copenhagen, Denmark

**Keywords:** mental health, young people, leisure, review, communities, wellbeing

## Abstract

**Introduction:**

A large proportion of young people reports poor mental health, which is a major public health concern. Positive mental health is important for young people's development, quality of life, functioning in everyday life, and long-term possibilities. Thus, there is a great need to develop and implement mental health-promoting initiatives and activities in young people's lives. Participating in organized leisure communities has a positive impact on mental health and wellbeing. However, more knowledge is still needed about *why* and *how* participating in organized leisure communities targeting young people can promote mental health. The aim of this study was to gain knowledge about the mental health-promoting potential of organized leisure communities for young people by exploring the *active ingredients* that contribute to mental health promotion.

**Method:**

Given the complexity of the subject, this study implemented a realist review approach to explore the interaction between context, mechanism, and outcome. The study follows Pawsons' five key steps for conducting a realist review: (1) clarify scope, (2) search for evidence, (3) study selection criteria, and procedures, (4) data extraction, and (5) data synthesis and analysis. The literature was systematically searched in the four databases PsycINFO, Scopus, Embase, and SocIndex.

**Results:**

In the literature search, a total of 11,249 studies were identified, of which 52 studies met the inclusion criteria. Based on the 52 studies, seven different contexts i.e., types of organized leisure communities for young peoples were identified. Across the seven different types of organized leisure communities, five active ingredients that promoted the mental health of young people were identified: social connectedness, development of skills, development of self-confidence, pleasure-driven participation, and safety and trust.

**Conclusion:**

This review contributes important knowledge about how to promote young people's mental health when participating in organized leisure communities. Moving forward, an important task consists of establishing and maintaining the five active ingredients in organized leisure communities through e.g., education and training that strengthens the skills and knowledge of those responsible for facilitating the leisure communities, such as sports coaches or music teachers, as these adults play a central role in supporting the active ingredients.

## 1 Introduction

Studies show that a large proportion of young people report poor mental health, which has become an alarming and pervasive trend worldwide (1, 2). The increasing prevalence of poor mental health among young people is a major cause of public health concern as it significantly impacts their development, quality of life, and functioning in everyday life. Furthermore, studies show that a low level of mental health and the development of mental illness can have serious consequences for young people's educational attainment and future employability (3). Additionally, poor mental health in the early years is associated with physical health problems and an elevated risk of developing mental illness in adulthood (4, 5). Thus, there is a great need to prioritize and protect initiatives and activities that promote positive mental health in young people's everyday lives.

Existing literature highlights the positive impact of participating in organized leisure communities on mental health and wellbeing (6, 7). Organized leisure communities can be characterized by predefined structures, including predetermined time, place, and duration of activities, standardized dress code (e.g., football uniforms), and adult monitoring and facilitation. However, these communities can manifest in various forms, encompassing different dimensions such as the type of activity (e.g., focusing on social, physical, and/or academic elements), dosage (frequency, intensity, and continuity), and the number of different activities that the people participate in (width) (8, 9). This multidimensional characteristic of organized leisure communities underscores the need for comprehensive knowledge in the field as existing literature often limits its investigations to specific contexts or target groups (10–13).

While previous studies have shed light on the relationship between extracurricular activities and academic performance as well as the protective role of leisure communities in negative social and behavioral outcomes (14–17), relatively fewer studies have examined the mental health-promoting potential of these communities. Notably, an umbrella review conducted in 2021 explored the effects of participation in organized leisure communities on the mental health of children and young people (18). However, the umbrella review highlighted the predominance of studies investigating mental health problems rather than mental health promotion and emphasized the need for further research in this area.

To address this knowledge gap, the aim of this study was to explore how participation in all types of organized leisure communities can have a mental health-promoting effect on young people. Through a comprehensive review of literature, the study sought to elucidate the mental health-promoting mechanisms inherent in organized leisure communities and the active ingredients that contributed to the promotion of young people's mental health and wellbeing. Specifically, this study aimed to answer the research question: What characterizes a mental health-promoting organized leisure community for young people?

## 2 Methods

We addressed the research question using a realist review approach. A realist review is developed as a method for literature studies with the aim of uncovering what works, for whom, and why, in what circumstances (19). It has its theoretical foundation in realism and focuses on exploring the interaction between context (C), mechanisms (M), and outcomes (O), rather than establishing causality between two conditions. In a realist review, the purpose is not to quantify a potential effect but to delve into the “why” and “how” questions within a research field. It aims to understand and explain how and why certain types of interventions work for specific target groups within particular contexts.

In this realist review, we examined the literature on mechanisms (M) in organized leisure communities (C) to explore how and why these types of leisure communities can be perceived as promoting mental health (O) for young people. This study followed Pawson's five steps for conducting a realist review (19). Although the steps are presented sequentially, the practical execution of the steps was overlapping and iterative, as anticipated in a realist review andhighlighted by Pawson et al. (19).

A protocol describing the methodological approach of the realist review was developed and published beforehand. The protocol was published on ResearchGate and Pure, a researcher portal at the University of Southern Denmark (20). For the sake of transparency, it is important to note any deviations from the procedure described in the protocol, which will be reflected upon in the subsequent sections.

### 2.1 Clarifying the scope of the review

**Figure 1:**
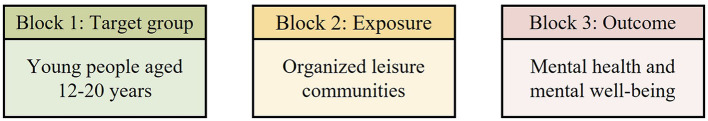
Search blocks.

To clarify the scope of the review, we used the PICo model (Population, Phenomena of interest, Context), a model developed to structure and define research questions in literature studies (21). Based on the PICo model, a search string was developed from three search blocks, where the target group, exposure, and outcome were delimited (Figure 1).

*Target group*: Young people were defined as people aged 12–20 years.

*Exposure*: Organized leisure communities were defined as communities in which young people participate in in their spare time, outside of school time, education, or (paid) spare-time job. Studies were included if the investigated population consisted of students, but only if the community in which the students participated occurred outside obligated school hours, implying participation based on their own engagement and will. We included studies in which the community was structured or organized, with a predefined framework for e.g., time, duration, and content of the community. Disorganized or unstructured leisure communities, such as “hanging out”, were excluded. Furthermore, online communities that emerged from social media or gaming were also excluded, as it was unclear to what extent they were organized according to the definition. Religious communities were also excluded because they likely contained elements of faith that are not comparable to other types of leisure communities. Organized leisure communities were further defined by consisting of an intersubjective community, constituted by more than one person.

*Outcome*: Mental health and mental wellbeing were defined as the positive aspects of mental health based on the Danish Health Authority's and WHO's definition of mental health “as a state of wellbeing in which every individual realizes his or her potential, can cope with the normal stresses of life, can work productively and fruitfully, and is able to contribute to her or his community.”(22). This encompasses various definitions and variants of positive mental health, such as life satisfaction, wellbeing, flourishing, quality of life, etc. The search excluded psychiatric diagnoses and symptoms of mental disorders such as anxiety, depression, and stress.

### 2.2 Search strategy

To further focus our research question and conduct the literature search and screening, we applied the following inclusion and exclusion criteria:

#### 2.2.1 Inclusion criteria

*Purpose:* Studies that investigated the characteristics of mental health-promoting organized leisure communities for young people.

*Target group*: Studies based on a population of young people aged 12–20 years. Studies that included a population but did not limit themselves to this age group were also included.

*Study design*: Studies employing qualitative, quantitative, and/or mixed methods.

*Language:* Studies published in Danish, Swedish, Norwegian, or English.

*Geography*: Studies that examined populations in the Western world,^1^ based on the definition from Statistics Denmark (23).

*Year of publication*: Studies published between July 2012 and July 2022.

*Document type:* Peer-reviewed studies published in scientific journals.

#### 2.2.2 Exclusion criteria

*Purpose:* Studies that exclusively examined characteristics of organized leisure communities related to the practical organization of the community (e.g., logistics or structural conditions), without addressing topics related to mental health promotion.

*Target group:* Studies that examined particular subgroups, such as specific patient groups. Studies exclusively focused on a target group below the age of 12 or above the age of 20, and studies that explored an adult population defined as +18 years, as this review focused on communities targeting young people.

*Document type:* Conference literature, books, book chapters, PhD dissertations, and opinion papers.

Deviating from the protocol: Language was included as an inclusion criterion when conducting the realist review, although the criteria was applied in the screening process and not in the literature search.

All types of reviews and meta-analyses that met the above criteria were included in the systematic literature search, as we applied a snowball method. This was done to ensure the inclusion of all relevant studies. However, reviews and meta-analyses were not included in the final analysis.

Development of the search string and search of literature was performed by specialized research librarians from the University of Southern Denmark. The literature was searched in the four databases: PsycINFO, Scopus, Embase, and SocIndex. Before the final literature search, a test search was conducted in May 2022 in Embase and Scopus, resulting in 13,383 studies after removing duplicates. Based on the test search, the inclusion criteria were supplemented with a delimitation of the year of publication to reduce the final number of hits.

### 2.3 Study selection, criteria, and procedure

The screening of literature was conducted in two phases, and a parallel screening phase for reviews and meta-analysis was carried out. The first and second authors conducted the two screening phases. The second author conducted the screening phase for reviews and meta-analyses, and relevant studies identified were also read in full text by the first author. In cases of disagreement, mutual discussion was employed to reach agreement.

*Screenings phase 1:* All studies identified based on the search string were imported into Covidence, a software program developed for organizing screening processes in literature studies. In Covidence, the first screening phase involved reading the title, abstract, and keywords if necessary.

*Screenings phase 2:* In the second screening phase, the full text of studies included from phase 1 was thoroughly read. Studies that complied with the inclusion and exclusion criteria of this study were included in the final cross-sectional analysis.

*Screening phase for reviews and meta-analyses:* All reviews and meta-analyses identified in the first and second screening phases were screened for relevant studies. Studies identified as potentially relevant were read in full text. If the studies met the inclusion and exclusion criteria, they were included in the final cross-sectional analysis.

### 2.4 Data extraction

**Figure 2:**
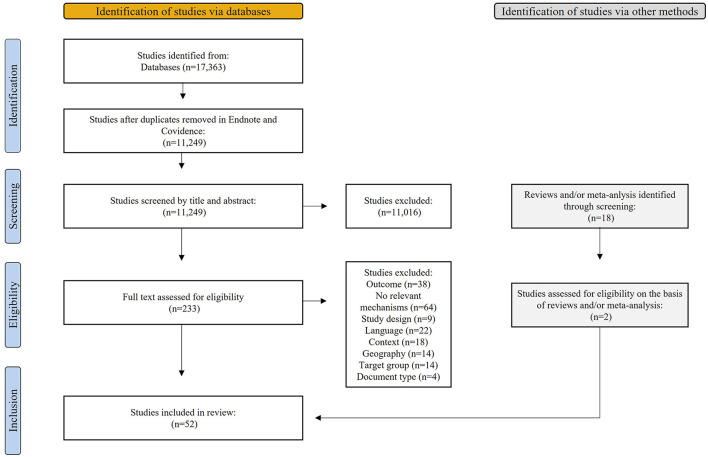
Flow chart.

In the search of literature, a total of 11,249 studies were identified after double-checking in EndNote and Covidence. Screening of titles and abstracts reduced the number to 233 studies, and reading the full texts further reduced it to 50 studies. During the screening of 18 systematic reviews and/or meta-analyses, two additional studies were identified, resulting in a total of 52 included studies for this review (Figure 2).

After the screening phases, we imported all the included studies into the qualitative software program NVivo, which was used as a tool to ensure analytic stringency. NVivo is particularly useful for handling large amounts of data and identify patterns (and thus knowledge gaps) across journal articles, which is why it is particular useful when conduction literature reviews (24). In NVivo, all 52 studies were coded according to a predetermined codebook that covered the following themes: nationality of the studied population, purpose of the study, study design and population size, examined target group including the age group of the population, type of organized leisure community, definition of mental health as outcome, and findings related to the aim of this review. Furthermore, based on the coding, we identified the context, mechanisms, and outcomes (CMOs) examined in each study cf. Section 2.5.

### 2.5 Data synthesis and analysis

After coding and identifying CMOs, a cross-sectional analysis was carried out by detecting patterns across the CMOs of different studies. This was done through three main steps: (1) The first and second authors reviewed approximately half of the 52 included studies each to identify the CMOs of the studies. To ensure uniformity in the two authors' identification of CMOs, a template was conducted beforehand in which context, mechanisms, outcomes, and the connections between mechanisms and outcomes had to be indicated. The template could be filled in by hand or digitally. After completing the templates, all studies were discussed jointly by the first and second authors to ensure agreement on the understanding of the studies' CMOs. (2) Based on the templates, it was now possible to categorize and divide the studies according to context. Then, by examining positive impact of investigated mechanisms on mental health outcomes within each context, it was possible to derive overarching themes within each context. (3) A cross-sectional analysis of the overall mechanisms/themes identified under each context was then undertaken across contexts.

## 3 Results

In total, we included 52 studies (Table 1), of which 25 were quantitative, 23 were qualitative, and four used mixed methods. Sixteen studies were categorized as intervention studies, as the studied organized leisure community (context) was not a continuously held activity independent of the study's investigation but rather an activity implemented for the purpose of the study.

**Table 1:** Included studies.

**References, Country**	**Population age (M*age*)**	**Organized leisure community (categorized context)**	**Design (*n* = sample size)**	**Mental health outcome (scale)**
Baker et al. (25), Australia	12–19 years (13.9)	Music (music)^*^	Qualitative (*n* = 85)	Wellbeing: feeling safe, having fun, pushing beyond boundaries
Battaglia et al. (26), Canada	18–19 years	Ice hockey (sport)	Qualitative (*n* = 10)	Athletic worth, sense of fun and enjoyment, interests in other activities, sport relationships
Bean et al. (27), Canada	10–18 years (15.4)	Several sports disciplines (sport)	Quantitative (*n* = 160)	Mental wellbeing (WEMWBS)
Calo et al. (28), Scotland	12–17 years	Music (music)^*^	Mixed method (*n* = 27)	Wellbeing (Good Childhood Index); self-confidence, trust, connectedness, engagement outside the intervention, engagement with the intervention
Cicognani et al. (29), Italy	16–26 years (20.8)	Voluntary/charity-, youth-, and religious groups (voluntary work and activism)	Quantitative (*n* = 835)	Social wellbeing (MHC-SF)
Clennon and Boehm (30), UK	Intervention 1: (13.0) Intervention 2: 12–18 years (12.0)	Musical program (creativity and art)^*^	Qualitative (intervention 1: *n* = 55) (intervention 2: *n* = 23)	Wellbeing: emotional awareness, self-esteem, anger management, self-esteem and confidence in sexual orientation
Conner et al. (31), US	Not specified (University students)	Activism (voluntary work and activism)	Qualitative (*n* = 42)	Mental health: social capital or connection to others, sense of purpose, effecting change, self-care and collective care
Cronin and Allen (32), UK	10–19 years (13.4)	Several sports disciplines (sport)	Quantitative (*n* = 202)	Psychological wellbeing: self-esteem (SDQII), positive affect (PANAS), life satisfaction (SWLS)
Dore et al. (33), Canada	16–39 years (18.5)	Several sport disciplines (sport)	Quantitative (*n* = 460)	Positive mental health (MHC-SF)
Duberg et al. (34), Sweden	13–18 years (16.0)	Dance program (dance)^*^	Qualitative (*n* = 112)	Finding embodied self-trust that opens new doors
Eriksen and Seland (35), Norway	14–20 years	Youth club (residual leisure communities)	Qualitative (*n* = 14)	Wellbeing: a safe place to be(long), social relations and identity formation, growth, purpose, and confidence
Forgeard and Benson (36), US	(19.0)	Athletic, academic, artistic, and prosocial activities (mixed leisure communities)	Quantitative (*n* = 512)	Psychological adjustment (CES-D, SPANE, DASS-21, PSWQ, SWLS, FS)
Gardner et al. (37), Australia	11–15 years (13.0)	Several sport disciplines (sport)	Quantitative (*n* = 313)	Enjoyment (SCM) and intention to continue
Geidne and Quennerstedt (38), Sweden	15–16 years	Several sport disciplines (sport)	Quantitative (*n* = 123)	What characteristics of a sports clubs make them feel good or bad, and what a sports club can do to make them stay as long as possible (open-ended questions)
Godfrey et al. (39), UK	8–18 years	Surfing (sport)^*^	Quantitative (*n* = 114)	Wellbeing (SCWBS)
González et al. (40), Spain	8–37 (14.7)	Football and basketball (sport)	Quantitative (*n* = 641)	Sport enjoyment (Intrinsic Satisfaction in Sport Questionnaire)
Gonzalez-Hernandez et al. (41), Spain	14–19 years (16.8)	Several sport disciplines (sport)	Quantitative (*n* = 436)	Psychological wellbeing (EBP)
Graupensperger et al. (42), US	(19.6)	Several sport disciplines (sport)	Quantitative (*n* = 697)	Wellbeing: life satisfaction (the happiness scale, the subjective health scale)
Harkins et al. (43), Scotland	6–16 years	Orchestra program (music)^*^	Qualitative (*n* = 125)	Mental and emotional wellbeing
Harris et al. (44), Australia	12–18 years	Hip hop dance program (dance)^*^	Qualitative (*n* = 171)	Psychological and social wellbeing
Hauseman (45), Canada	10–18 years	Youth-led community arts hubs (creativity and art)^*^	Qualitative (*n* = 27)	Perspective on program structure, youth participation and qualities of effective youth leaders and program coordinators
Hignett et al. (46), UK	13–16 years (14.3)	Surfing program (sport)^*^	Mixed method (*n* = 58)	Wellbeing (BPHS-Y)
Jakobsson and Lundvall (47), Sweden	9–19 years	Several sport disciplines (sport)	Qualitative (*n* = 114)	Views of participation in sport
Jakobsson (48), Sweden	15–19 years	Several sport disciplines (sport)	Qualitative (*n* = 18)	What make teenagers participate
Jetzke and Mutz (49), Germany	(23.2)	Several sport disciplines (sport)	Quantitative (*n* = 4,698)	Life satisfaction (SWLS)
Kinoshita et al. (50), Canada	(15.4)	Several sport disciplines (sport)	Quantitative (*n* = 196)	Thriving: intention to continue sport, athletic subjective wellbeing (PANAS, SWLS), goal progress
Laurence (51), UK	15–17 years	Club participation (mixed leisure communities)^*^	Quantitative (*n* = 7,970)	Life satisfaction (how satisfied are you with your life on a scale from 1 to 10?)
Leversen et al. (52), Norway	15–16 years	Different leisure activities (mixed leisure communities)	Quantitative (*n* = 3,273)	Life satisfaction (Huebner's SLSS)
Light and Yasaki (53), Australia & Japan	13–16 years	Basketball (sport)	Mixed method (*n* = 12)	Sport enjoyment and experience
Lindgren et al. (54), Sweden	18–25 years	Several sport disciplines (sport)	Qualitative (*n* = 55)	How sport clubs retain young adults
Merati et al. (55), Canada	7–12 years	Music program (music)^*^	Qualitative (*n* = 8)	Wellbeing: emotional-, social-, personal-, and educational wellbeing
Moreau et al. (56), Canada	(17.6)	Sportsprogram (sport)^*^	Qualitative (*n* = 9)	Psychosocial benefits
Navickas et al. (57), Lithuania	18–23 years	Volunteer work (voluntary work and activism)	Quantitative (*n* = 200)	Life quality
Oberle et al. (58), Canada	(9.2–12.3)	Different types of extracurricular activities (mixed leisure communities)	Quantitative (*n* = 10,149)	Life satisfaction (SWLS—adapted for Children)
Parker (59), US	Not specified (high school)	Choir (music)	Qualitative (*n* = 40)	Self-growth
Phillips Reichter and Weiss (60), US	11–14 years (12.9)	Sport and music (mixed leisure communities)	Quantitative (*n* = 366)	Motivational orientation (subscales of the MOSS)
Price and Weiss (61), US	15–18 years (15.9)	Soccer (sport)	Quantitative (*n* = 412)	Psychosocial and team outcomes: perceived competence (SPPA), intrinsic motivation (MOSS), enjoyment, team cohesion (GEQ), collective efficacy (CEQS)
Reverberi et al. (62), Italy	14–20 years (16.2)	Soccer (sport)	Quantitative (*n* = 415)	Psychological wellbeing (Ryff's PWB Scale)
Rottensteiner et al. (63), Finland	14–15 years	Several sport disciplines (sport)	Quantitative (*n* = 1,936)	Sport enjoyment (enjoyment scale)
Scrantom and McLaughlin (64), Nothern Ireland	11–15 years	Dance program (dance)^*^	Qualitative (*n* = 10)	Psychosocial benefits
Sebire et al. (65), UK	11–12 years	Dance program (dance)^*^	Mixed methods (*n* = 571)	Perceived level of exertion and enjoyment
Stark and Newton (66), US	15–18 years (16.3)	Different dance environments (dance)	Quantitative (*n* = 84)	Psychological wellbeing: positive and negative affect (PANAS), body-esteem (BESAA), friendship (IPPA)
Stevens et al. (67), Australia	8–14 years (10.0)	Circus (residual leisure communities)	Qualitative (*n* = 55)	Wellbeing
Super et al. (68), the Netherlands	11–17 years	Several sport disciplines (sport)	Qualitative (*n* = 22)	Sports experiences
Swann et al. (69), Australia	12–17 years (14.7)	Several sport disciplines (sport)	Qualitative (*n* = 55)	Mental health
Tamminen et al. (70), Canada	(16.3)	Several sport disciplines (sport)	Quantitative (*n* = 451)	Sport enjoyment (Scanlan's measure of enjoyment) and commitment
Van Hoye et al. (71), France	8–14 years	Football (sport)	Quantitative (*n* = 342)	Enjoyment (enjoyment subscale from the Intrinsic Motivation Inventory), drop-out intentions, self-esteem (PSDQ), perceived health (single item)
Vella et al. (72), Australia	12–19 years (14.6)	Several sport disciplines (sport)	Quantitative (*n* = 383)	Wellbeing (Key's MHC-SF)
Vettraino et al. (73), Canada	Not specified (7th and 8th grade)	Theater (creativity and art)^*^	Qualitative (*n* = 29)	Wellbeing
White et al. (74), Australia	(14.4)	Several sport disciplines (sport)	Qualitative (*n* = 144)	Positive affect
Wright et al. (75), Canada	14–20 years (16.7)	Arts program (creativity and art)^*^	Qualitative (*n* = 32)	Positive development
Yuriev (76), Canada	Not specified (University students)	Volunteer work (voluntary work and activism)	Qualitative (*n* = 5)	Satisfaction

^*^Organized leisure community as an intervention.

Included studies were based on populations from 16 different countries. The majority of study populations were from Canada (*n* = 12), followed by Australia (*n* = 7) and the USA (*n* = 7). In the 52 included studies, the age of the studied population was reported in various ways, such as mean age or age groups. In addition, we included four studies that did not indicate age but provided the grade level of the population in school. Among the included studies, the lowest examined age was 6 years, and the highest 39 years. However, most of the studies focused exclusively on young people aged 12–20 years.

### 3.1 Identified organized leisure communities

Among the 52 studies, we categorized the context into seven different types of organized leisure communities: sport, dance, music, creativity and art, and voluntary work and activism. Furthermore, the category “Mixed leisure communities” covered studies that did not limit themselves to examining one specific leisure community, and the category “Other leisure communities” covered studies where the studied context differed significantly from all the other studies.

#### 3.1.1 Sport

In total, 27 studies concerned young people's participation in sport (26, 27, 32, 33, 37–42, 46–50, 53, 54, 56, 61–63, 68–72, 74). Through the analysis of their CMOs, four central mental health-promoting mechanisms were identified within sports communities: positive social relations and cohesion with teammates, learning and development of skills, pleasure-driven participation, and inclusive and competent coaches (Table 2). Among these studies, 17 explored participation across various sport disciplines, while 10 focused on specific sport disciplines, such as hockey or football. Furthermore, three studies were classified as intervention-based.

**Table 2:** Sport.

**Studies**	**Definition of the context: sport**	**Identified mechanism**
*N* = 27 (26, 27, 32, 33, 37–42, 46–50, 53, 54, 56, 61–63, 68–72, 74)	Covers studies that examined participation in several different sports, one specific sport such as hockey, or an intervention consisting of a sport activity.	• Positive social relations and cohesion with teammates • Learning and development of skills • Pleasure-driven participation • Inclusive and competent coaches

Certain studies did not utilize a standardized outcome measure for positive mental health but rather measured sport enjoyment as an outcome (37, 60, 63, 70, 71). These studies were incorporated into the analysis due to the close resemblance between the definitions and measurements of sport enjoyment and mental health within the context of sports. Sport enjoyment can be conceptualized as “a positive affective response to the sports experience, that reflects generalized feelings such as pleasure, liking, and fun” (77).

The reviewed studies highlighted positive social relations and the experience of cohesion with teammates as influential in promoting mental health within sports communities (38, 47, 53). Two studies underscored the importance of young people deriving pleasure from togetherness with friends and feeling a sense of belonging within the community (48, 74). A study inferred that physical activity in a social context tends to foster mental health more effectively than individual sport participation (33). Another study suggested that a team-focused approach, emphasizing support and improvement over competition and conflicts, was more likely to enhance young people's experience of sport enjoyment (70). The importance of fulfilling young people's basic needs for autonomy, competence, and relatedness—core principles of the self-determination theory by Deci and Ryan (78)—was further reinforced by studies examining these needs within sports communities (27, 40).

Learning and development of skills were identified as additional mechanisms promoting mental health in sports communities (48, 53, 74). It was found that challenging competitions and games, in conjunction with engaged trainers, played a significant role in motivating young people and fostering their skill development (47). The role of competence development as a mental health-promoting factor was also corroborated by studies exploring the self-determination theory (27, 41).

Several studies also pointed out that participation in sport must be fun and pleasure-driven for it to have a mental health-promoting effect. This was confirmed by the studies exploring self-determination theory (49, 63, 72). Two studies both underlined that participation in sport should stem from the young people's own desire to promote mental health (38, 74).

The role of the coach emerged as central in sports communities for young people (68, 69), acting as a significant figure to connect with (37, 38). The coach was recognized for their responsibility to support and promote an environment that emphasized e.g., inclusion, fair play, and respect in the game (71). Additionally, it was deemed essential for the coach to possess particular skills, such as special management behavior (61), the capability to initiate initiatives that foster personal and social skills (32), and the importance of avoiding behavior that might be perceived as degrading (26).

#### 3.1.2 Dance

Five studies explored the relationship between dance and mental health and wellbeing (34, 44, 64–66). Dance, perceived both as a sport and a leisure activity, was associated with positive effects on young people's wellbeing, highlighting three principal mechanisms: the development of self-confidence, the establishment of positive social relations, and the facilitation of emotional and creative expression (Table 3). Four of the five studies focused on dance interventions.

**Table 3:** Dance.

**Studies**	**Definition of the context: dance**	**Identified mechanism**
*N* = 5 (34, 44, 64 –66)	Covers studies that examined participation in dance.	• Development of self-confidence • Positive social relations • Facilitation of emotional and creative expression

The studies suggested that dance can enhance self-confidence and self-belief among young people. Participating in rehearsals and performances was associated with a bolstered self-confidence (64), and engagement in dance interventions corresponded with an increase in self-confidence in various life aspects (65). Dance also offered young people a platform for self-acceptance, fostering belief in their abilities, and promoting a positive self-image (34). Engaging in specific dance styles, such as hip-hop, was reported to displace negative thoughts with a sense of empowerment, thereby improving self-confidence and self-perception (44).

Three studies delved into the role of positive social relations within the context of dance. It was found that diversity within dance interventions cultivated positive relationships and friendships among young participants (44, 64). Collaborations focusing on hip-hop dance between different schools also facilitated the establishment of new friendships (44). Moreover, young people reportedly formed friendships with peers perceived as different, consequently positively affecting their wellbeing (64). The fostering of solidarity and new friendships contributed to a sense of belonging, enhancing the mental health of young dancers further (34).

Two studies emphasized that dance served as an avenue for emotional and creative expression, particularly among vulnerable girls (34, 44). These girls exhibited a broad spectrum of emotions through dance, both positive and negative (34). The potential for emotional and creative expression through dance was underscored as a crucial mechanism for self-expression and the unleashing of creativity (44).

#### 3.1.3 Music

Five studies investigated the effects of participation in music communities on young people's mental health (25, 28, 43, 55, 59). The studies highlighted three central mechanisms contributing to young people's wellbeing and mental health: the development of self-confidence, the cultivation of positive social relations and a sense of belonging, and the experience of a safe community (Table 4). Four out of these five studies were intervention-based.

**Table 4:** Music.

**Studies**	**Definition of the context: music**	**Identified mechanism**
*N* = 5 (25, 28, 43, 55, 59)	Covers studies that examined participation in music leisure communities in the form of choirs, playing an instrument or songwriting.	• Development of self-confidence • Positive social relations and a sense of belonging • The experience of a safe community

In four of the studies examined, engagement in musical activities was highlighted as a significant mechanism through which young people could develop musical competencies, thereby fostering a sense of self-confidence and pride in their achievements (25, 43, 55, 59). Notably, performance and demonstration of their musical talents were associated with significant boosts in self-esteem. Furthermore, the process of pushing personal boundaries and consistently working to refine their musical skills often led to accomplishments that engendered a sense of pride, contributing to increased self-confidence (25).

In three of the studies, a sense of community, positive social relations, and a feeling of belonging emerged as crucial elements within musical engagements (43, 55, 59). Participating in collective music-making activities provided young people with a unique sense of belonging, while simultaneously fostering the establishment of new friendships and a shared sense of unity.

Additionally, three of the studies underscored the importance of experiencing a sense of safety within the musical community as a vital aspect impacting wellbeing (25, 28, 43). These studies indicated that music communities offer young people a secure environment to express themselves freely, without fear of judgment. This sense of safety could be further reinforced by the presence of dedicated music tutors and the establishment of trusting relationships between the young people and adults within the community (28, 43).

#### 3.1.4 Creativity and art

Four studies were identified that examined the impact of youth engagement in diverse creative and artistic communities on young people's wellbeing and mental health (30, 45, 73, 75). These activities often culminated in final products, performances, or exhibitions. The findings highlighted three key mechanisms: artistic development, relationship building and trust, as well as competence and self-confidence (Table 5). Three out of the four studies employed intervention methodologies.

**Table 5:** Creativity and art.

**Studies**	**Definition of the context: creativity and arts**	**Identified mechanism**
*N* = 4 (30, 45, 73, 75)	Covers studies that examined participation in different forms of creative activities, such as theater, musicals or handicrafts that often lead to a product, performance or showing.	• Artistic development • Relationship building and trust • Competence and self-confidence

Three studies highlighted the significance of skill acquisition and competence development in various creative activities, placing particular emphasis on the evolution of the young peoples' artistic identities (30, 45, 75). The improvement of skills within their chosen art forms was found to positively influence the wellbeing of the young people. Over time, many participants gradually cultivated an artistic identity, largely through exploration and engagement with new art forms. This process enhanced their self-confidence and solidified their self-perception as artists. Remarkably, one study indicated that the enhancement of artistic skills could extend its influence beyond self-perception, affecting aspects such as the behavior and fashion choices of some participants (30). Furthermore, participants' engagement with art persisted beyond the completion of the intervention programs in one of the studies, suggesting that art became a lasting element of their identities (75).

The theme of relationship building, and trust was prevalent across all studies. Personal growth within the programs fostered mutual recognition and appreciation among participants (45). The formation of relationships and friendships within these programs had a positive impact on the participants' mental health and wellbeing. Remarkably, several participants maintained these new friendships even years after completing the programs (75). The role of program facilitators was emphasized in three of the studies, with participants highly valuing their support, skills, and approachability (30, 73, 75).

#### 3.1.5 Voluntary work and activism

Four studies investigated the impact of participation in voluntary work, including activism, on the mental health of young people (29, 31, 57, 76). The activities typically took place within educational environments and involved engagement with non-governmental organizations (NGOs) or politically activist student groups. Three core mechanisms were identified as contributing to the positive mental health and wellbeing outcomes experienced by youth engaging in voluntary work: skill development, fostering of positive social relations and a sense of belonging, as well as the experience of assisting others and effecting change (Table 6).

**Table 6:** Voluntary work and activism.

**Studies**	**Definition of the context: voluntary work and activism**	**Identified mechanism**
*N* = 4 (29, 31, 57, 76)	Covers studies that examined participation voluntary work, charity work or activism. This can be in the form of involvement in Non-Profit Organizations or politically activism students' groups.	• Skill development • Positive social relations and sense of belonging. • Experience of assisting others and effecting change

In all studies, social relations and a sense of belonging were significant factors for mental health and wellbeing. Participation in voluntary and charitable organizations, youth groups, and religious organizations were shown to enhance social wellbeing and foster a sense of community. The opportunity to meet new people, feel connected with others, and form friendships further amplified the positive experiences associated with engaging in voluntary work (29, 31, 57, 76).

Two studies highlighted the acquisition and enhancement of new skills as a significant aspect of young peoples' experience of voluntary work. The ability of young people to develop their skills within the context of voluntary work was found to contribute to their positive experiences (57, 76).

A unique and meaningful aspect of voluntary work, highlighted in two studies, was the sense of making a difference and aiding others (31, 76). This mechanism was characterized by the young people's experience of engaging in actions that have tangible, beneficial impacts on their communities or causes they care about. It encapsulates not only the act of helping others but also the satisfaction and personal fulfillment derived from knowing that their efforts contribute to broader social change.

#### 3.1.6 Mixed leisure communities

Five studies examined the impact of various leisure activities on the mental health and wellbeing of young people (36, 51, 52, 58, 60). These activities encompassed creative pursuits, sports, and music. The analysis revealed three key mechanisms: self-determination and autonomy, experience of competence, and positive social relations and sense of belonging (Table 7). One study was an intervention.

**Table 7:** Mixed leisure communities.

**Studies**	**Definition of the context: mixed leisure communities**	**Identified mechanism**
*N* = 5 (36, 51, 52, 58, 60)	Covers studies that examined different types of leisure communities such as creative activities, sport, and music.	• Self-determination and autonomy • Experience of competence • Positive social relations and sense of belonging

Three studies investigated the impact of self-determination and autonomy on young people (36, 51, 52). The findings suggest that perceived control is crucial in enhancing life satisfaction, as observed in an intervention program (51). Furthermore, a correlation between mastery and wellbeing is evident in sports and academia, while this relationship is not observed in activities associated with creativity and prosocial behaviors (36). However, one study did not identify a mediating effect of autonomy on the relationship between leisure activities and life satisfaction (52).

Three studies explored mechanisms related to the experience and development of competence (36, 52, 60). One study found that competence served as a mediator between leisure activities and life satisfaction (52). Another study highlighted the link between perceived competence and enjoyment in sports (60). The third study examined creative self-efficacy as a mechanism, which explained the relationship between variables and wellbeing in prosocial activities but not in creativity, sports, and academia (36).

Across four studies, the significance of social relations and attachment in the context of mixed leisure activities was explored (51, 52, 58, 60). These studies emphasized the importance of peer attachment, a sense of connectedness, the role of various social resources, and the influence of positive friendship quality on enjoyment within sports and music communities.

#### 3.1.7 Other leisure communities

Two studies on circus training and youth club activities were categorized as “other leisure communities” as they did not fit categorically within the remaining studies (35, 67). Despite the apparent differences between these two types of leisure communities, the analysis revealed two transversal mechanisms: the development of self-confidence and the significance of positive social relations (Table 8).

**Table 8:** Other leisure communities.

**Studies**	**Definition of the context: other leisure communities**	**Identified mechanism**
*N* = 2 (35, 67)	Covers studies that examined other leisure communities in the form of circus training and participation in a youth club.	• Development of self-confidence • Positive social relations

Exploring circus trainings impact on young people's wellbeing it is underscored that the circus training made the young people feel proud and brave contributing to the development of self-confidence (67), and that participation in youth clubs promoted the development of competencies contributing to self-assurance (35). Furthermore, positive social relations emerged as central components in both contexts. Peer support and guidance assumed a significant role within the circus training setting (67), while youth clubs offered an optimal environment for establishing social networks and nurturing positive relationships with adult mentors (35).

### 3.2 Active ingredients

The preceding sections have recounted the mental health-promoting mechanisms examined within seven distinct types of organized leisure communities. This section will present mechanisms found to be common across the categorized leisure communities and the 52 incorporated studies, i.e., the active ingredients that promoted young peoples' mental health when participating in organized leisure communities.

**Figure 3:**
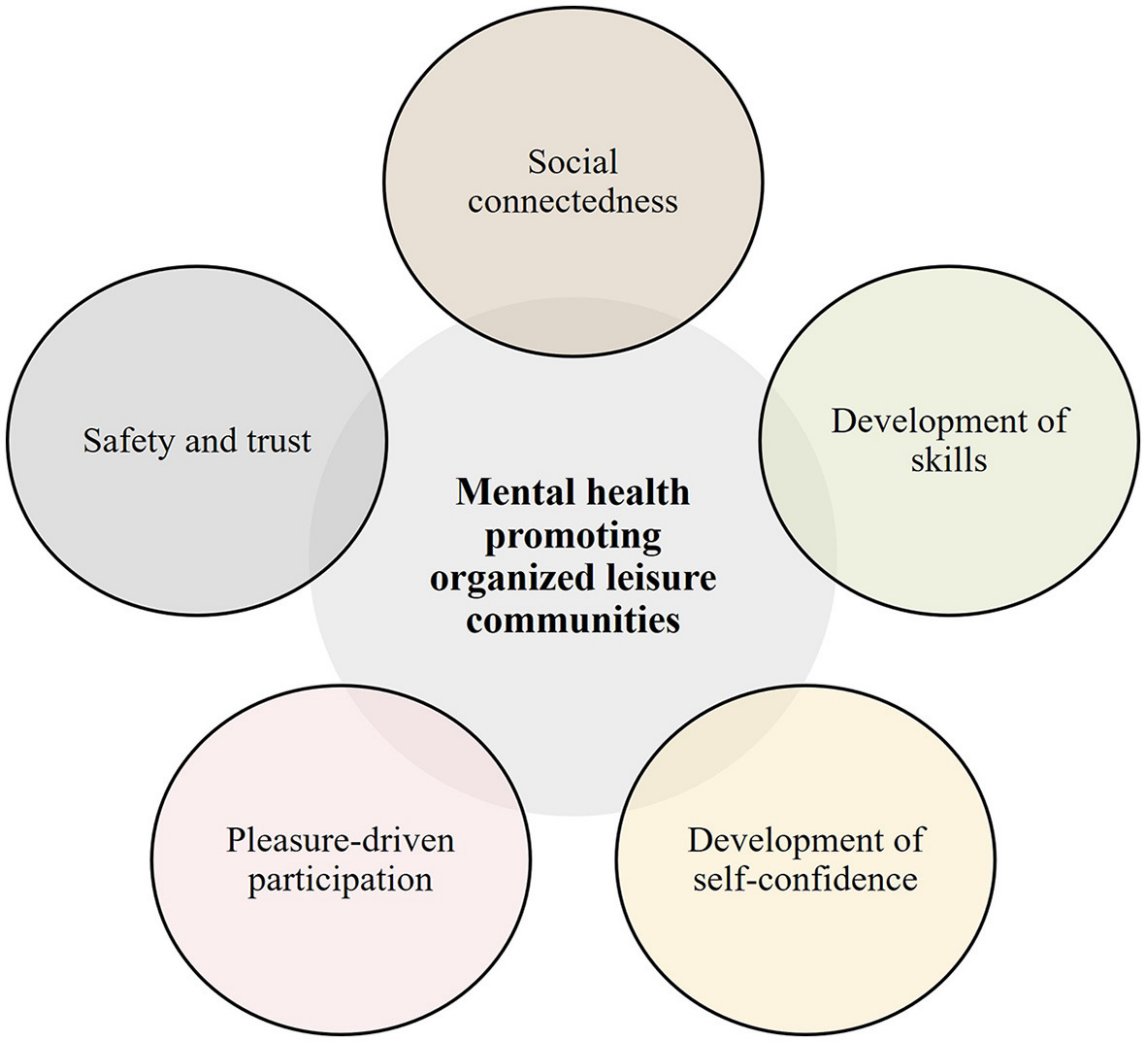
The five active ingredients.

As illustrated in Figure 3, five active ingredients were identified: social connectedness, development of skills, development of self-confidence, pleasure-driven participation, and safety and trust.

Social connectedness was the most salient active ingredient across all contexts. Many of the included studies affirmed the importance of friendships, experience togetherness, and positive relations with peers and adults within the leisure community for young people's wellbeing. Social connectedness extended beyond mere community involvement; it suggested a profound relational attachment and sense of belonging. In several studies, it was clear that new friendships often originated from participation in leisure communities, and the experience of these positive relations was critical for deriving pleasure from participation.

Development of skills was further identified as an active ingredient. The experience of advancing and acquiring the necessary skills for the activity tended to motivate young people through the provision of a sense of success. Through challenging tasks within the leisure activity, young people had opportunities to push boundaries and grow.

Development of self-confidence was notably a mental health-promoting active ingredient in creative leisure communities such as dance, music, and art. In these leisure communities, it seemed critical for the young people to build confidence and self-belief in their ability to contribute to the activity with their skills (self-efficacy).

Pleasure-driven participation was an active ingredient evident within sports context but also applied to the “Mixed leisure communities” category. Numerous studies emphasized the importance of young people participating in leisure communities driven by personal desire rather than external expectations or needs.

The experience of a safe and trusting community, in the relation between young people and between young people and the adults involved in the community (e.g., music teachers or sports coaches), was another salient active ingredient. To promote their mental health, it was vital for young people to perceive the leisure community as safe and trusting, a place where they could truly be themselves and where they felt accepted by the peers and the adults.

These five active ingredients were prominent across the 52 studies; however, some were more prevalent in specific contexts than others. For instance, some active ingredients had a greater overlap in artistic communities such as music, dance, and art. Moreover, the active ingredients should be understood as overlapping and mutually reinforcing. For example, the experience of developing competencies and skills could enhance young peoples' self-confidence. Demonstrating competencies through performances or exhibitions could also foster a stronger sense belonging and experience of cohesion within the leisure community. Likewise, a safe and trusting environment could lay the ground for the development of positive social relations, and these positive social relations could in turn aid young people in building their self-confidence.

## 4 Discussion

This review systematically examined the literature to identify mental health-promoting active ingredients in organized leisure communities. The analysis revealed five active ingredients that enhance young people's positive mental health and wellbeing: social connectedness, development of skills, development of self-confidence, pleasure-driven participation, and safety and trust. Social connectedness was the most prominent ingredient across seven different types of leisure communities. The review also underscored the significance of skill development and self-confidence, which were interlinked factors. The role of self-efficacy was emphasized, particularly within creative leisure communities, where the young people's belief in their capabilities positively impacted their wellbeing and engagement. Pleasure-driven participation and experience with a safe, trustful environment facilitated by adult figures, such as music instructors or coaches, were identified as vital for the wellbeing of young people.

However, prevalent theories on mental health determinants show considerable overlap with these five active ingredients, reflecting both individual-level factors (e.g., “positive sense of self”), social-level factors (e.g., “supportive social relationships”), and structural-level factors (e.g., “safe environments”) (79). This suggests that the active ingredients may be relevant not only in the context of leisure communities but also in other youth contexts, such as educational environments. This points to a need for future research to investigate further.

Despite the common occurrence of gender segregation in organized leisure communities—especially in sports—the majority of the studies included both boy and girl participants, suggesting that the active ingredients benefit both genders in promoting mental health. Seven of the included studies focused solely on girls (34, 40, 53, 59, 61, 65, 66), and three on boys (62, 69, 72). However, only two of these studies included gendered aspects of the leisure community as an analytic perspective (59, 69). Thus, this review points to a need for a further examination of the gender perspectives to potentially uncover differences in boys' and girls' experiences. For instance, findings from previous research indicates that motivations for participating in sports can differ by gender (80, 81), where one study concludes that boys, to a greater extent notify the joy of competition, popularity, and the desire to become a sports star, as the basis of the motivation compared to girls (81). Likewise, another study points to the “masculine character” of sports as a factor for young girls' continued desire to participate in sport (82). Further research on this could inform more tailored and effective strategies to enhance the mental health and wellbeing of young people in organized leisure communities, ensuring inclusivity and addressing potential gender-specific needs.

The crucial role of facilitators within organized leisure communities emerges as an important finding of this review. Coaches, mentors, and facilitators of leisure communities do more than merely guide activities; they can create environments and atmospheres where young people can flourish. These facilitators play a critical role in establishing community norms and conduct standards, significantly influencing participant experiences, and directly impacting overall wellbeing. They can also encourage individual strengths, contributing to the development of self-confidence and skills among young people. These findings are supported by insight within the sport literature that examines how coaches, through knowledge and behavior, can promote the mental health of athletes. For example, a qualitative study concludes that coaches see themselves as an important resource for identifying concerns, facilitating help, and promoting engagement in sports, but that greater mental health literacy would boost the coaches' skills to promote the athletes' mental health (83). Additionally, recent reviews call for qualified programs to equip coaches with the necessary competencies to promote the mental health of athletes (84, 85). Thus, going forward an important task consist of establishing educational programs for those responsible for leisure communities—sport coaches, art teachers, or art instructors—to provide them with the skills, tools, and knowledge to support and initiate the active ingredients within their leisure community.

### 4.1 Strengths and limitations

This realist review provides novel insights into the active ingredients promoting mental health within organized leisure communities for young people. However, certain limitations should be acknowledged. Addressing these in future research will contribute to a more comprehensive understanding of the factors influencing mental health within organized leisure communities.

Firstly, the review lacks a comprehensive exploration of the multidimensional nature of organized leisure communities. Most reviewed studies did not consistently consider various dimensions of the organized leisure community such as activity type, dosage, and width of participation. A nuanced understanding of these aspects could provide additional insights into the factors influencing mental health benefits within different leisure community contexts. Additionally, this reviews' focus on the positive impacts of organized leisure communities on mental health may have overlooked potential negative aspects or challenges associated with participation in these communities. Exploring potential adverse effects or challenges could provide a more balanced understanding of the overall impact of organized leisure communities on mental health and wellbeing.

Secondly, the absence of a methodological quality assessment or weighting of the included studies limits the ability to assess the overall quality and robustness of the results. Conducting a rigorous quality assessment could provide valuable insights into the reliability and validity of the findings. For example, the preponderance of cross-sectional designs among the quantitative studies identified restricts the establishment of causal relationships. Longitudinal or experimental designs would offer stronger evidence for establishing causal links between participation in leisure communities and mental health outcomes. Moreover, most identified intervention studies either relied on qualitative designs or lacked control groups in quantitative studies, diminishing the ability to draw definitive conclusions about the effectiveness of mental health-promoting organized leisure communities for young people. More intervention studies with rigorous designs and control groups could strengthen the evidence base and enable a more robust evaluation of these interventions' impact on mental health outcomes. Thus, a more in-depth analysis of the methods, quality, and findings of both the excluded and included studies could have strengthened this review further.

Despite these limitations, we note several strengths of this study. The methodological strengths of the systematic review include using a realist approach, allowing an in-depth examination of the context, mechanisms, and outcomes related to the mental health-promoting potential of organized leisure communities for young people. By focusing on the underlying mechanisms and contextual factors, the review provides a deeper understanding of how and why these studies may influence positive mental health outcomes for young people. The realist approach is particularly valuable for capturing the complexity and multifaceted nature of the studies, shedding light on the underlying processes contributing to the observed positive impacts. The comprehensive literature search strategy, developed and executed by specialized research librarians, ensured thorough and systematic identification of relevant studies, minimized the risk of publication bias, and increased the inclusion of diverse perspectives and findings. Involving two independent researchers in the screening process provided additional quality assurance, reducing the potential for errors and enhancing the reliability of the included studies.

Regarding the included studies, the review covers a wide range of methodologies and data sources, enabling a comprehensive analysis of the topic. The inclusion of both qualitative and quantitative studies provides a more holistic view of the mental health-promoting organized leisure communities for young people, thus strengthening the validity and reliability of the review's findings.

## 5 Conclusion

This realist review provided valuable insights into the mental health-promoting potential of organized leisure communities for young people. The study identified five key active ingredients: social connectedness, development of skills, development of self-confidence, pleasure-driven participation, and a safe and trusting environment, which significantly contribute to young people's mental health and wellbeing. The findings emphasize the pivotal role of facilitators in creating inclusive and supportive environments within these communities. However, the study also acknowledged limitations, such as the lack of a comprehensive exploration of various dimensions within these communities and called for further research to address these gaps and enhance the evidence base. Overall, this review highlights the importance of organized leisure communities in enhancing the mental health and wellbeing of young people, potentially guiding the development of targeted interventions and policies across diverse leisure settings.

## Author contributions

AK: Conceptualization, Formal analysis, Investigation, Methodology, Writing—original draft. TU: Formal analysis, Investigation, Writing—original draft. AF: Conceptualization, Funding acquisition, Supervision, Writing—review & editing.

## Footnotes

^1^Andorra, Australia, Belgium, Bulgaria, Canada, Cyprus, Denmark, Estonia, Finland, France, Greece, the Netherlands, Ireland, Iceland, Italy, Croatia, Latvia, Liechtenstein, Lithuania, Luxembourg, Malta, Monaco, New Zealand, Norway, Poland, Portugal, Romania, San Marino, Switzerland, Slovakia, Slovenia, Spain, the United Kingdom, Sweden, the Czech Republic, Czechoslovakia, Germany, Hungary, the United States, the Vatican City, and Austria.
